# The role of imaging in endodontics

**DOI:** 10.1038/s41415-025-8511-z

**Published:** 2025-04-11

**Authors:** Wisam Sarsam, Jonathan Davies, Samira K. Al-Salehi

**Affiliations:** 711282015843411494285 Institute of Dentistry, Queen Mary University of London, Turner Street, London, E1 2AD, United Kingdom; 863362328820065833101 Department of Dental Radiology, Guy´s and St Thomas´ NHS Foundation Trust, London, SE1 9RT, United Kingdom

## Abstract

Dental radiography is an essential tool in endodontics for determination of diagnosis, treatment planning and monitoring of treatment outcome. Conventional two-dimensional imaging remains the most commonly used and the standard method of radiographic imaging in endodontics due to accessibility and low radiation exposure. The use of cone beam computed tomography is increasing worldwide due to the benefits of three-dimensional visualisation of the teeth under investigation and surrounding structures. Its use, however, should be considered on a case-by-case basis, taking into consideration the benefits and increased dose of radiation in line with published guidelines.

## Key points

- Dental imaging is a fundamental tool in endodontics, used for diagnosis, treatment planning and review of endodontic outcomes.
- Intra-oral periapical radiographs remain the most common imaging modality used in endodontics and should be used in conjunction with electronic apex locators.
- Low-volume cone beam computed tomography (CBCT) provides 3D information on root morphology and surrounding structures. Due to the higher radiation dosage relative to conventional dental imaging techniques, CBCT is normally used for the more complex endodontic cases.

## Introduction

Dental radiography is essential in endodontics for diagnosis, treatment planning and monitoring outcomes. Currently used imaging modalities in endodontic practice include intra-oral periapical (IOPA) radiography and cone beam computed tomography (CBCT). The IOPA is the most commonly used imaging technique in endodontics due to accessibility (availability and cost) within the dental surgery, simplicity (training and reporting) and low dose of radiation. CBCT is an important imaging modality in endodontic practice, with enhanced benefits in both diagnosis and treatment planning.^1^ This paper aims to summarise the applications of radiology in endodontics, with a specific focus on intra-oral radiographs and CBCT imaging.

## Dental radiography in endodontic diagnosis and treatment planning

**Fig. 1:**
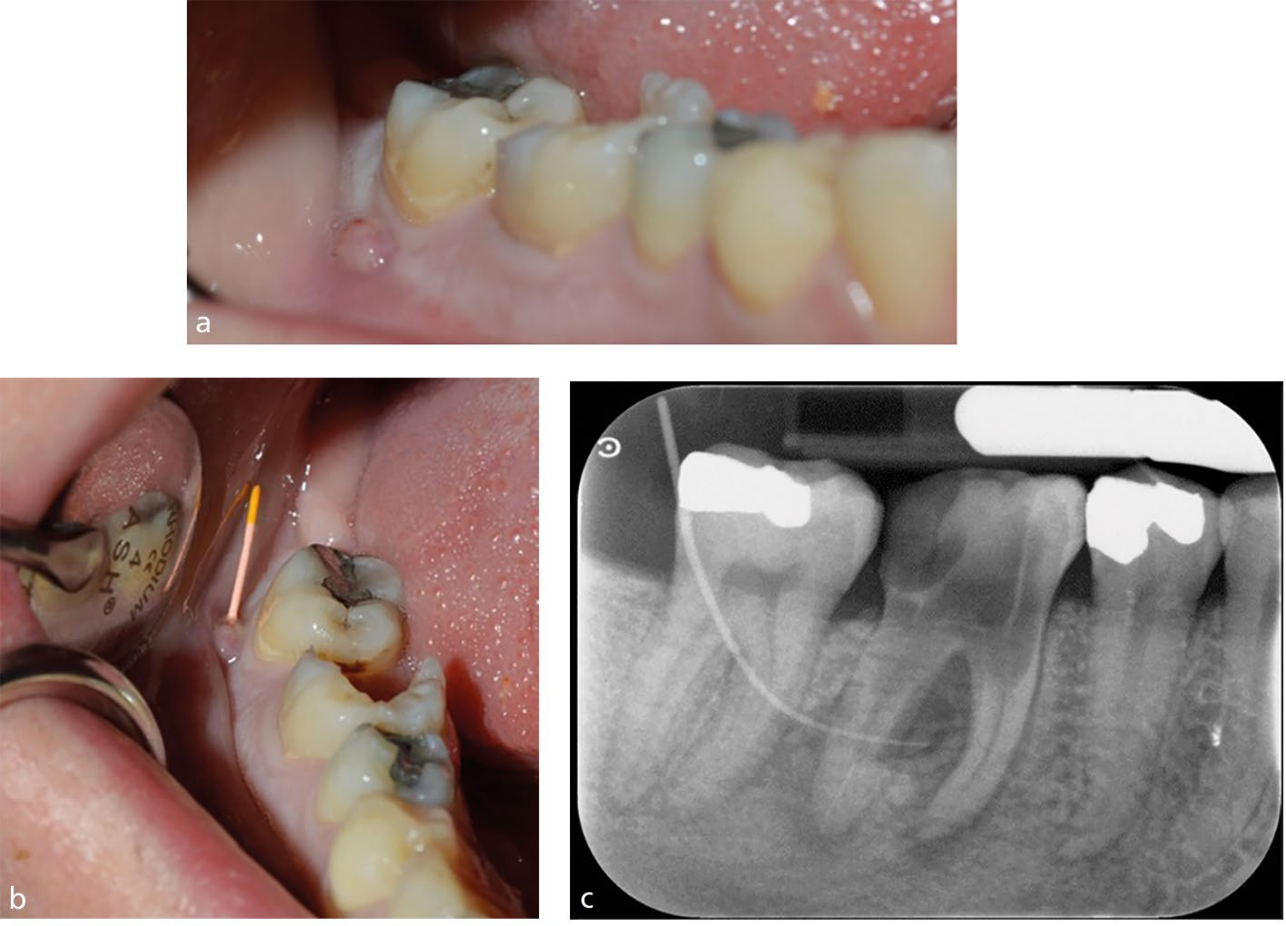
a) Discharging sinus adjacent to the lower right second molar 47 (responsive to cold testing). b) Size 20 gutta-percha point inserted into the sinus. c) IOPA of the image in (b) showing the gutta percha point tracking to the peri-radicular lesion of the lower right first molar (46)

The standard imaging technique in diagnosis and treatment planning in endodontics is an IOPA radiograph, which should be taken following a dental history and a clinical assessment to justify the exposure. The ideal periapical radiograph should be taken using a paralleling technique to display optimal image geometry of the tooth in question and at least 3 mm of periapical bone to allow adequate assessment of the apical status. IOPA radiographs provide essential information about the pulp, root canal anatomy and the peri-radicular tissues that may contribute to the diagnosis and treatment planning. For example, in Fig. 1a, where a discharging sinus in relation to a chronic endodontic infection was detected adjacent to the lower right second molar, a gutta-percha point (Fig. 1b) was inserted to trace the sinus before exposing the radiograph. Occasionally, a draining sinus may present some distance away from the infected tooth; therefore, the gutta-percha point will trace the sinus back to the original source of infection. Loss of continuity of the lamina dura, and development of a periapical radiolucency as seen in Fig. 1c, are typical radiographic signs of apical periodontitis.

**Fig. 2:**
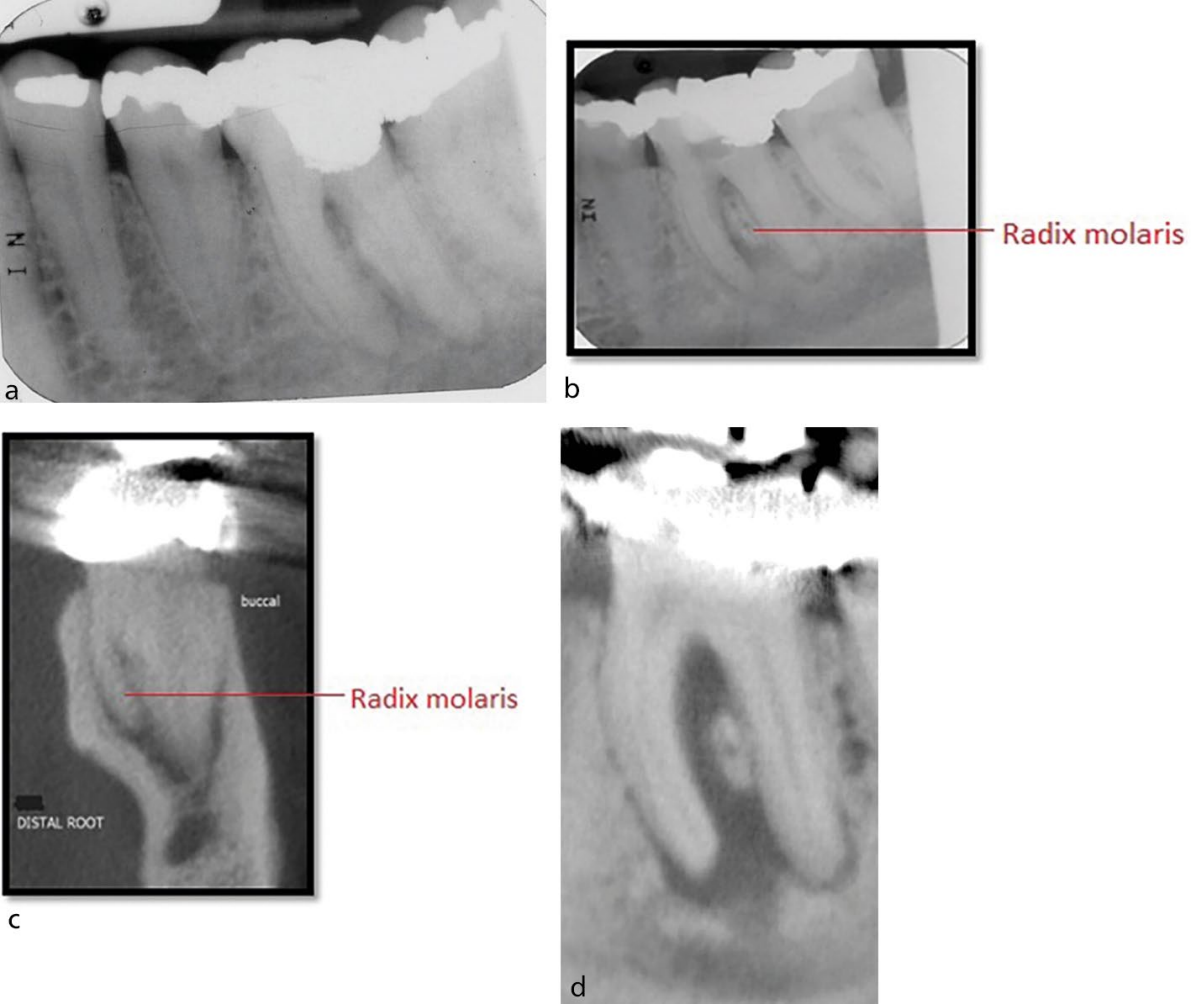
a, b) Showing a lower left first molar (36) using paralleling technique and an angled IOPA radiograph revealing evidence of a radix molaris. More information was required before commencing endodontic treatment in this case and hence a low volume CBCT of the 36 was taken which confirmed the complex anatomical features. c, d) Coronal view and sagittal view

**Fig. 3:**
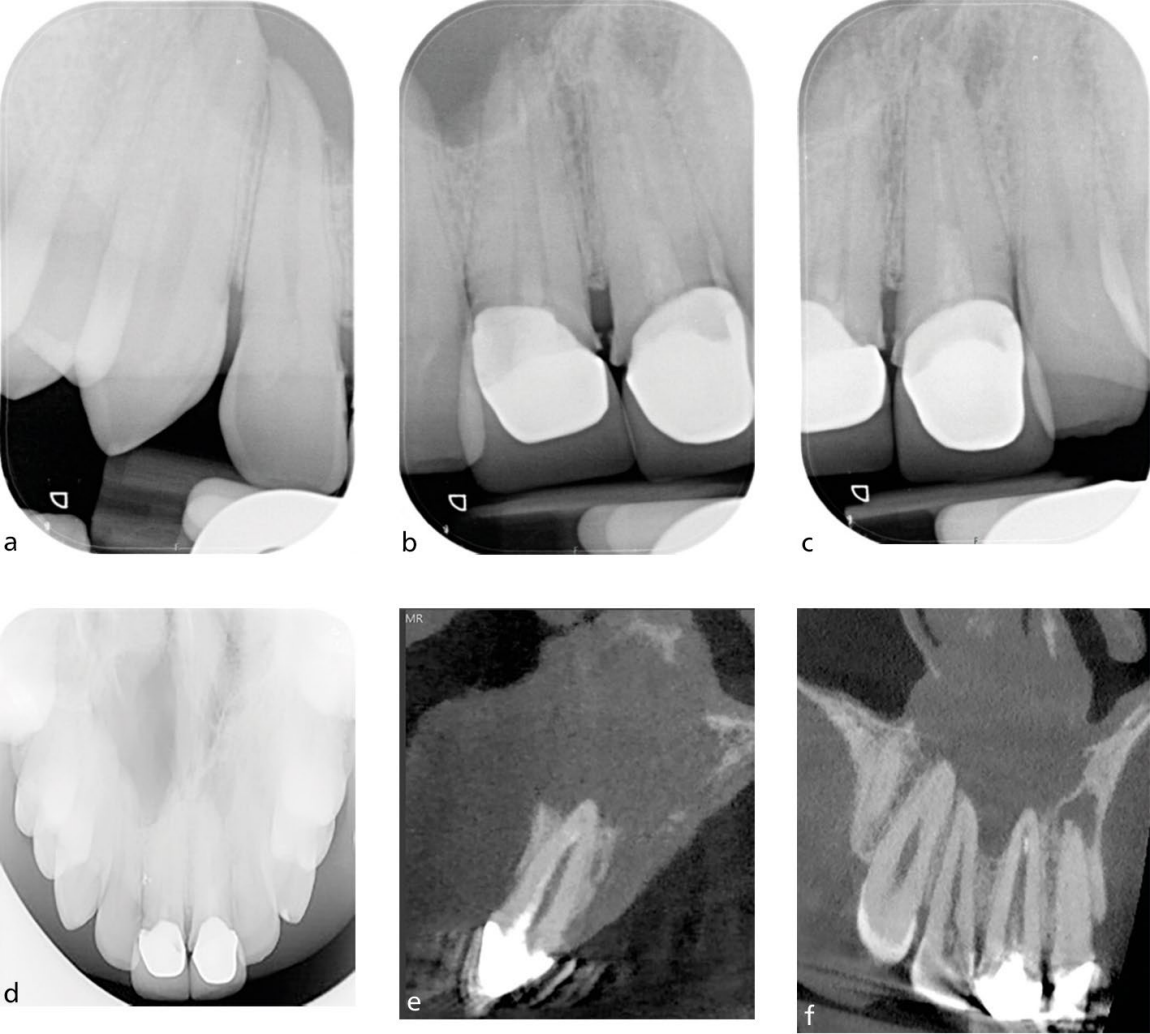
a, b, c) Series of periapical radiographs showing radiolucency related to apices of the 11, 12, and 13. d) Maxillary occlusal radiograph showing a large non-corticated radiolucency in relation to the apices of the 11, 12, 13, and 14 in a patient with a history of trauma to the anterior teeth. This case was of a complex nature requiring surgical intervention and a CBCT was needed for planning purposes. e, f) The CBCT images, sagittal view and coronal view clearly demonstrate the extent of the lesion

IOPA radiographs remain the standard and first line radiographic investigation of an endodontically involved tooth. This is due to their relatively low dose of radiation, relatively high image resolution and accessibility in terms of cost, size of equipment, and technique simplicity (training) and interpretation (reporting). In addition to the paralleling technique, for teeth with multiple roots and/or canals, a parallax rule involving a second image with the x-ray tube shifted in a 10-20 degree in the mesial or distal horizontal angle can be used to determine the root canal anatomy.^2^ This may allow the separation of the root canals and eliminate overlap of anatomical structures. Figure 2 illustrates the parallax technique revealing complex anatomy. A vertical shift may also be used, for example, when taking an upper anterior occlusal radiograph. This view may assist in the diagnosis of dental trauma in detecting oblique fractures which, depending on the x-ray beam angulation, may not be visible on an IOPA. It may also be useful for the detection of unerupted teeth and large apical lesions, the full extent of which may not be fully visible on an IOPA (Fig. 3).^3^ In specialist and hospital settings, however, occlusal views are becoming superseded by ‘small field of view' CBCT. The majority of cases seen in such tertiary referral centres are of a complex nature, often requiring 3D imaging.

Advanced imaging may be required when a diagnostic yield from a conventional dental radiograph does not provide sufficient useful information. CBCT is an advanced imaging system that provides visualisation of the dentition and the surrounding anatomical structures in three dimensions. This comes at an increased cost to the patient in terms of radiation, the provider in terms of surgery space plus equipment and maintenance fees, and the operator in terms of additional training in taking, interpretating and reporting on 3D images. Table 1 provides a comparison of effective doses from different dental radiographs compared to one day of background radiation.^4^^,^^5^ As a part of the justification process, the clinician must consider the potential benefits in diagnosis and treatment planning when using CBCT versus the risks of increased radiation exposure complying with the ALADAIP (As Low As Diagnostically Acceptable being Indication-orientated and Patient-specific) principle.^6^ The European Academy of Dentomaxillofacial Radiology recommends two levels of training for clinicians involved in the application of CBCT.^7^ Level 1 (core course): intended to train dentists in the skills required to justify and prescribe a CBCT examination and understand the reported images; and Level 2: a more advanced level, including interpreting and reporting on CBCT images. The European Society of Endodontology (ESE) issued a position statement on the use of CBCT in endodontics.^8^ It recommended a field of view (FOV) of <5 x 5 cm focused on the area of interest in selected cases, following a detailed clinical examination, including a conventional radiograph. A small FOV reduces the level of radiation exposure, as well as streak- and beam-hardening artefacts, which may affect the quality of the image.

**Table 1:** Comparison of effective dose from different dental radiographs and one day background radiation^4^^,^^5^

**Radiation**	**Effective dose μSV**
Background radiation/day in the UK	7
Intra-oral x-ray	5
Panoramic	20
CBCT small	148
CBCT medium	177
CBCT large	212

**Fig. 4:**
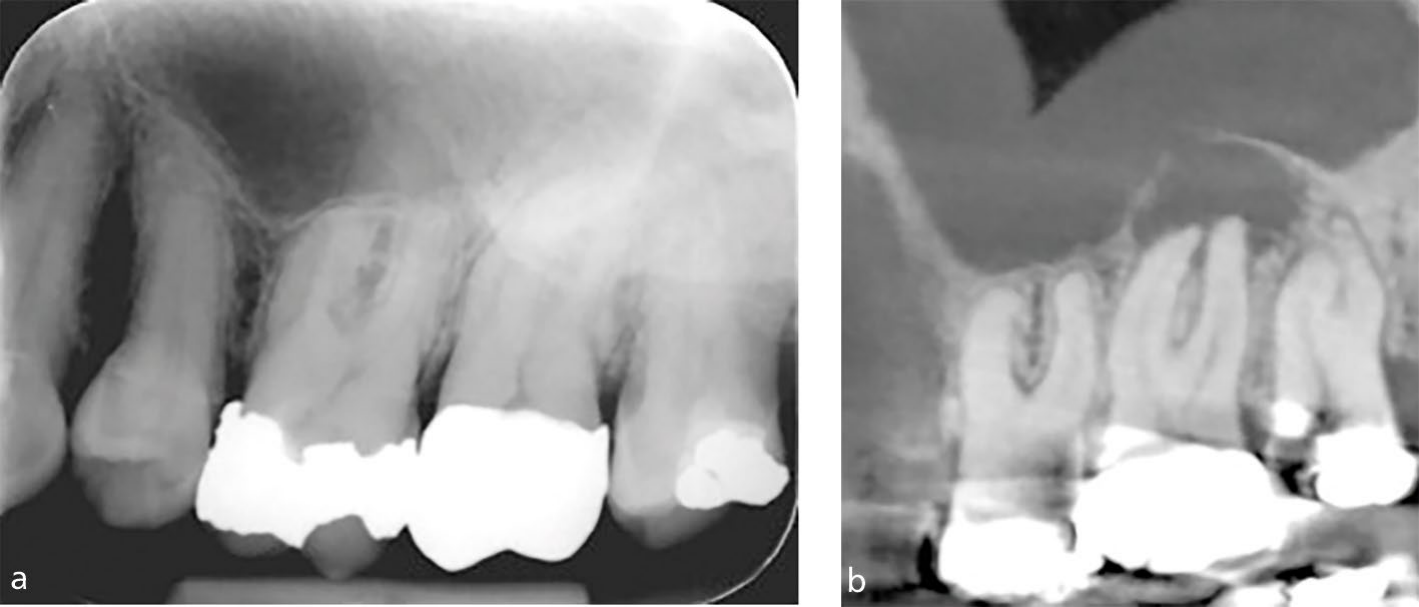
Patient presented with symptoms of apical periodontitis with clinical tenderness to percussion from all three extensively restored molars. No other clinical signs of inflammation were detected and pulp testing was inconclusive. a) The periapical radiograph did not show any clear evidence of pathology; the area of interest is masked by the zygomatic arch. b) A small FOV CBCT revealed a large periapical radiolucency associated with the 27

CBCT has been found to be more accurate than IOPA radiographs in detecting periapical radiolucencies.^9^ Superimposition of anatomical structures may hide early signs of periapical radiolucency, leading to difficulties in diagnosis, for example, when clinical signs and symptoms are not consistent.^10^^,^^11^ CBCT helps overcome these challenges by providing a volume of the FOV which can be analysed in different planes, allowing earlier diagnosis, treatment and potential improved prognosis (Fig. 4). A clinical study reported that 28% more periapical lesions were detected with CBCT compared to IOPA radiographs.^12^

**Fig. 5:**
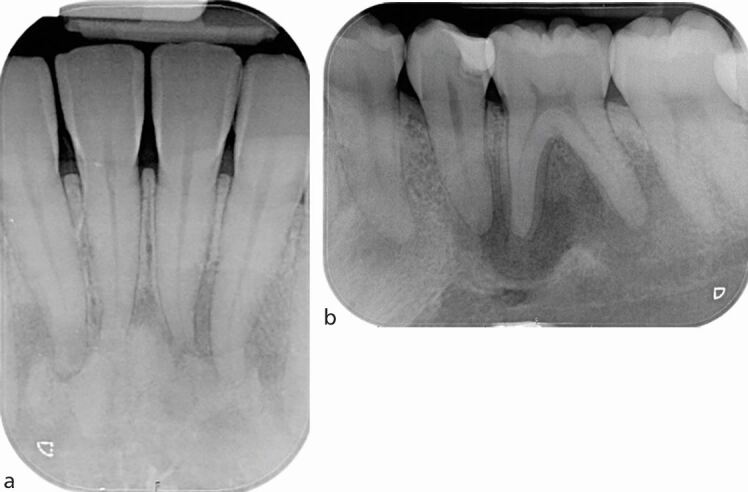
a) IOPA radiograph of lower incisors showing a mixed radiolucent-radiopaque appearance of the lesion located at the apices of the lower anterior teeth. The lamina dura associated with the 31, 41 and 42 are lost. All teeth tested positive with sensibility testing and a diagnosis of FCOD made. Endodontic treatment is not indicated. The teeth are vital. b) IOPA radiograph of the 35 and 36 (same patient as Figure 5a) showing a large radiolucent area in relation to apices of 35 and also 36. The 35 distal restoration has secondary caries. Both teeth tested positive with sensibility testing, further confirming a diagnosis of FCOD. Endodontic treatment is not indicated. The teeth are vital

It is important that radiographic findings are used in conjunction with the patient's history of symptoms and clinical examination, for example, sensibility testing when making a diagnosis. Figure 5 shows a case of florid cemento-osseous dysplasia (FCOD). All teeth tested positive with both cold and electrical pulp testing, and endodontic treatment was, therefore, not indicated. Instead, the patient was kept under review; if the teeth become necrotic, endodontic treatment may become necessary.^13^

**Fig. 6:**
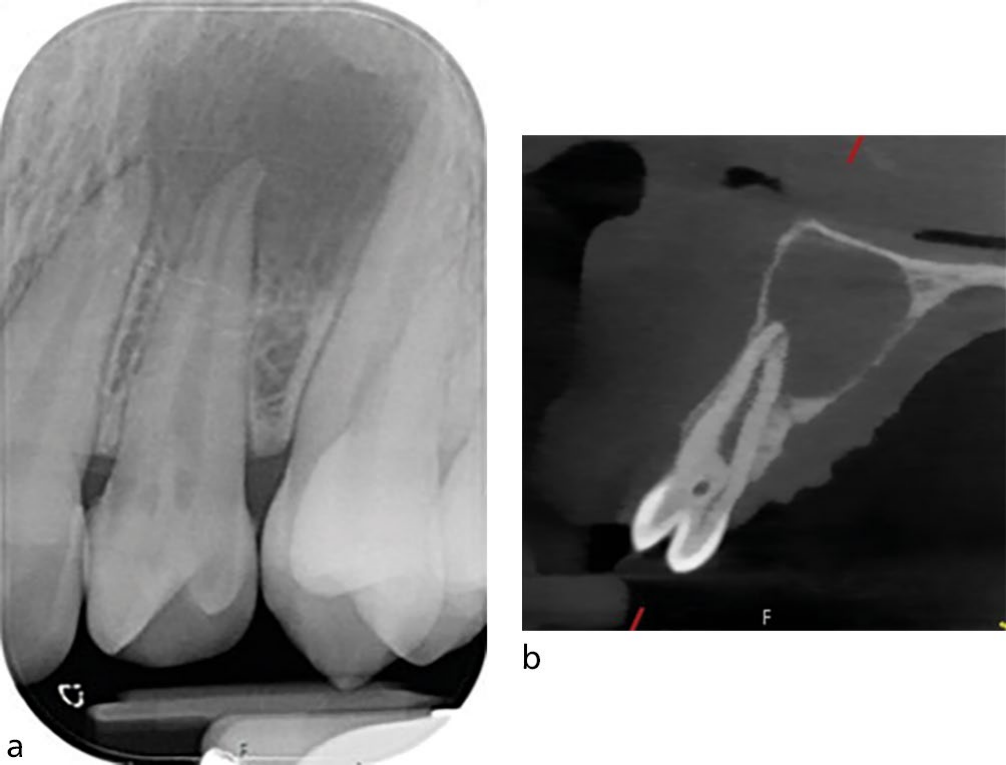
a) IOPA showing the 22 with a dens invaginatus (Oehlers' Classification Type I) and talon cusp. b) Sagittal view from CBCT of the same tooth showing the full extent of the lesion

CBCT 3D imaging provides more information on the teeth with complex anatomy, such as those with a dens invaginatus (Fig. 6).

**Fig. 7:**
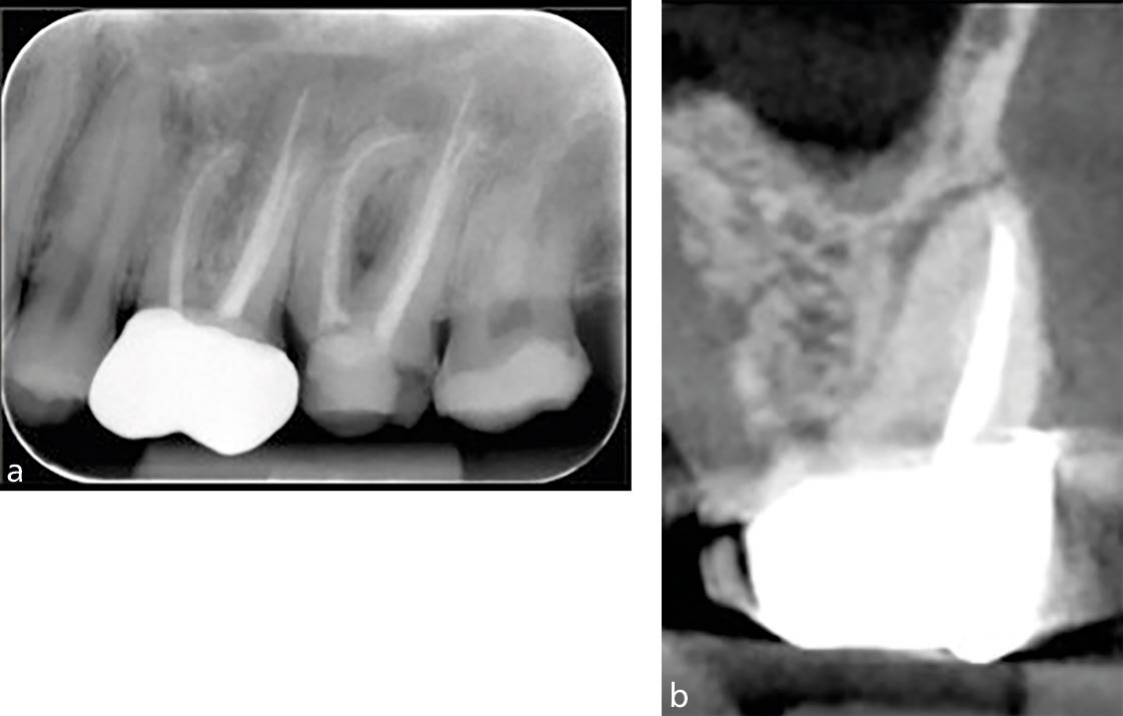
Patient presented with symptomatic upper left first molar (26), with tenderness on palpation above the mesio-buccal root. a) IOPA radiograph of endodontically treated 26 and 27. b) CBCT coronal view of the mesio-buccal root of the 26 showing the root canal filling is off centre, untreated MB2, and periapical radiolucency

CBCT is also more accurate than IOPA radiographs in identifying missed anatomy (Fig. 7). It has been reported that only 8% of second mesiobuccal (MB2) canals were identified using conventional radiographs, compared to 54% with CBCT imaging.^14^

**Fig. 8:**
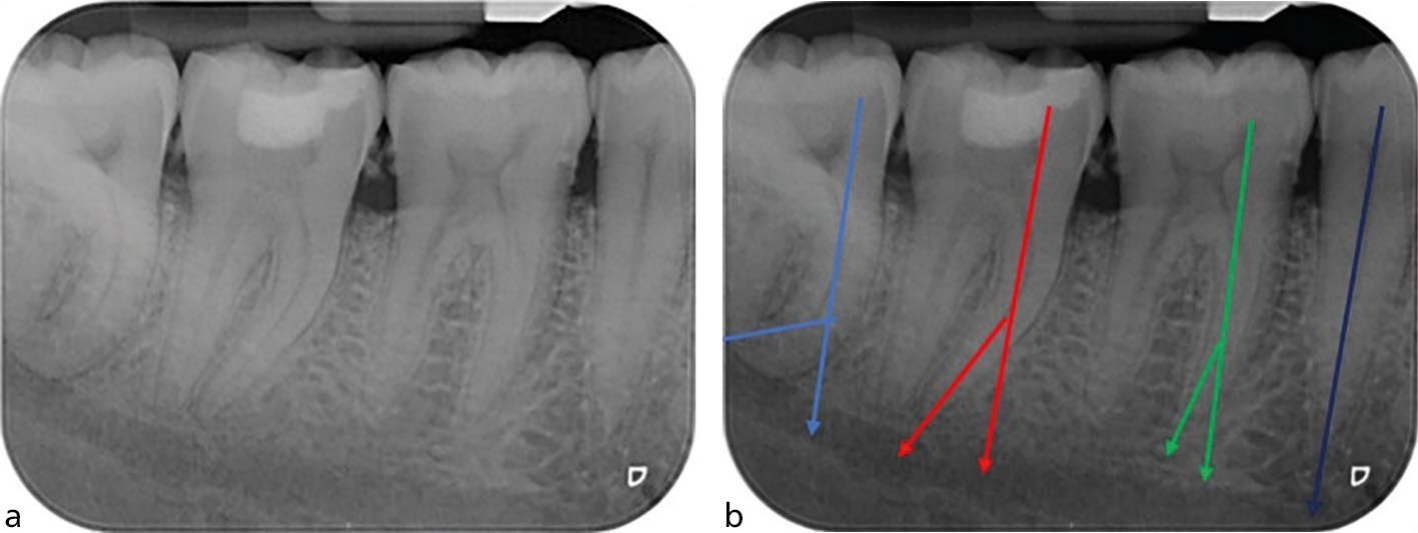
a) Periapical view of the lower right second premolar, first second and third molars. b) Estimation of canal curvature, left to right; extreme (light blue), moderate(red), slight (green) and straight canal (dark blue). The angle is formed at the intersection of the two lines

IOPA radiographs can also be used to determine the complexity of curvature of canals through measurement of root angulation, which can be determined by drawing a line passing through the apical one-third of the canal and the long axis of the tooth (Fig. 8). Canal morphology can be classified as slight or no curvature (<10°), moderate curvature (10-30°), or extreme curvature (>30°) based on the American Association of Endodontists' Endodontic case difficulty assessment form and guidelines.^15^

**Fig. 9:**
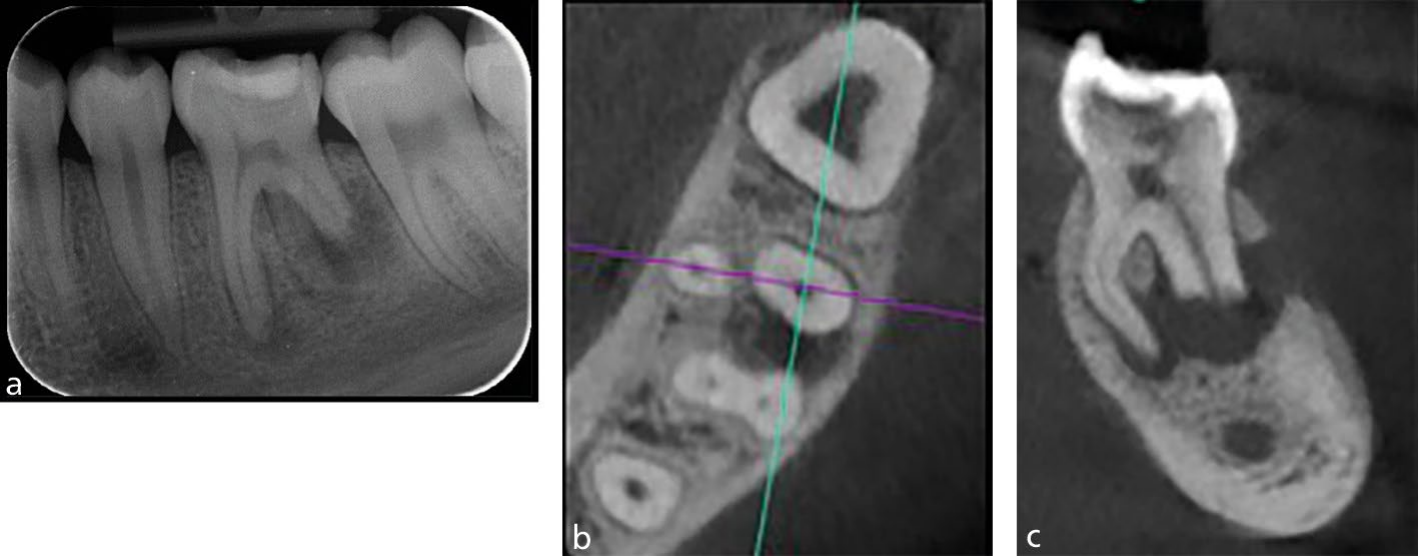
a) IOPA radiograph showing inflammatory resorption of the distal root of the 36 and periapical radiolucency of mesial and distal canals. b) CBCT axial slice showing the extent of the lesion, perforating the buccal cortical plate. c) Coronal view revealing severe curvature of the disto-lingual canal in the bucco-lingual plane which is not evident on the IOPA radiograph

**Fig. 10:**
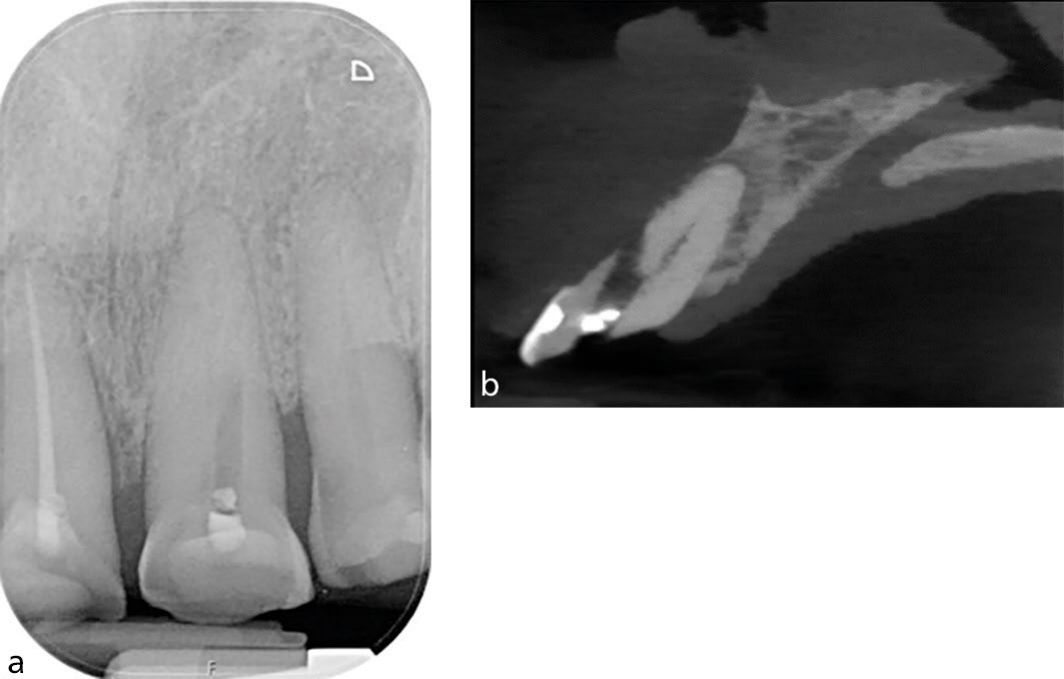
a) IOPA showing an attempted access of a calcified canal, tooth 21. b) CBCT sagittal view of the tooth showing a large labial perforation rendering the tooth non-restorable

**Fig. 11:**
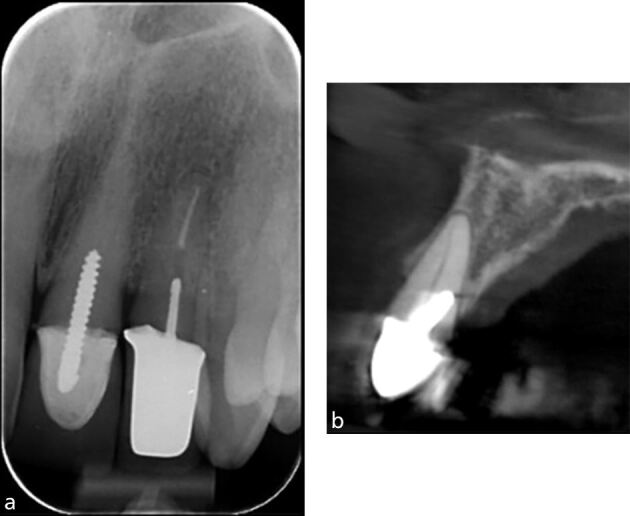
a) IOPA of the 21 with a threaded post which appears to be well-aligned in the mesial and distal planes. b) CBCT sagittal view of the same tooth showing the post is misaligned with near palatal perforation. Note the beam-hardening artefact mimicking a fracture line

CBCT provides information in the bucco-lingual plane that cannot be provided by an IOPA 2D periapical radiograph, for example, ‘hidden' curvatures (Fig. 9), perforations (Fig. 10) and post alignments (Fig. 11). Note the presence of scatter radio-opaque and beam-hardening radiolucent artefacts may affect image interpretation and diagnosis (Fig. 11a).

**Fig. 12:**
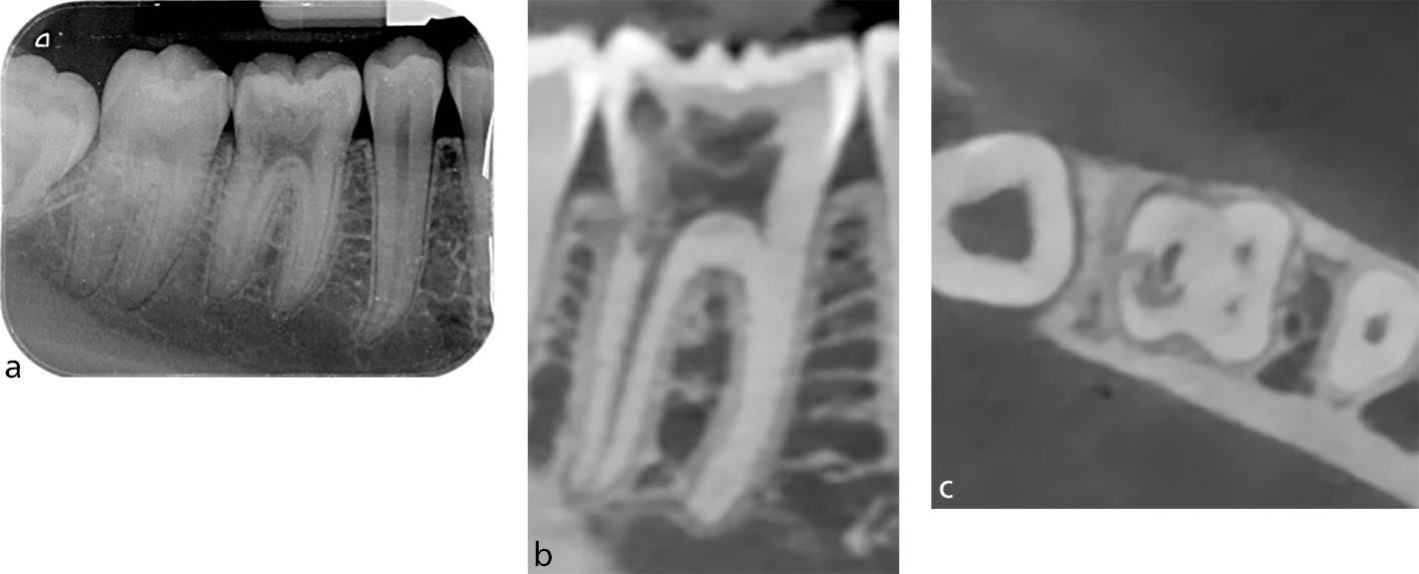
a) IOPA of the 46 showing signs of ECR. b) Sagittal CBCT section. c) Axial CBCT section of the 46 revealing extensive cervical resorption with portal of entry located in the distal interproximal region. Patel *et al*.^17^ classification: 2CP. Surgical access and treatment poses a significant risk to the adjacent tooth and therefore, it was planned for an extraction

**Fig. 13:**
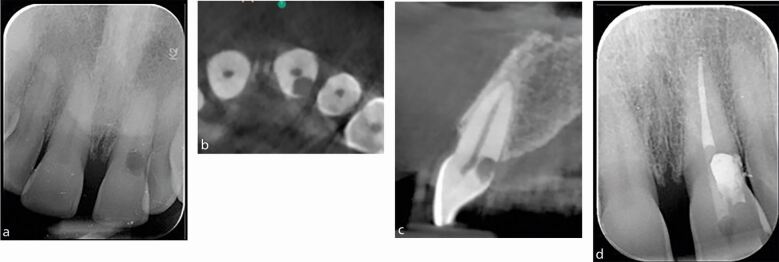
a) The 21. Resorption detected on IOPA. b) Axial CBCT section showing the extent of circumferential spread. c) CBCT sagittal section reveals the apical extension in relation to the marginal bone, and proximity to the pulp. d) Endodontic treatment and surgical repair of the perforation was completed. Patel *et al*.^17^ classification: 2BP

The extent of root resorption is often underestimated using conventional 2D imaging, particularly when the lesion extends deep in the bucco-palatal/lingual dimension. For example, it may be difficult to determine the origin and the true nature of the type of resorption without identifying the portal of entry, which may not be visible on a periapical radiograph (Fig. 12, Fig. 13). Classification systems used for external cervical resorption (ECR) can help to determine prognosis and treatment of these lesions.^16^ A more recent classification of ECR is based on 3D images^17^ and in addition to the pulpal involvement and depth, also considers the circumferential spread of the lesion.

CBCT may reveal a pattern of bone loss along a vertical root fracture (VRF) which otherwise may not be visible on a IOPA radiograph, particularly if the bone loss is along the buccal or lingual/palatal surface. However, unless there is some displacement of root fragments, a crack will not be visible radiographically. Furthermore, beam-hardening radiolucency and scatter due to streak artefacts associated with radio-opaque materials, such as root fillings or metallic posts (Fig. 11b), may be misinterpreted as fracture lines. Therefore, at present, there is insufficient evidence to advocate the use of CBCT in routine detection of VRFs.^18^

**Fig. 14:**
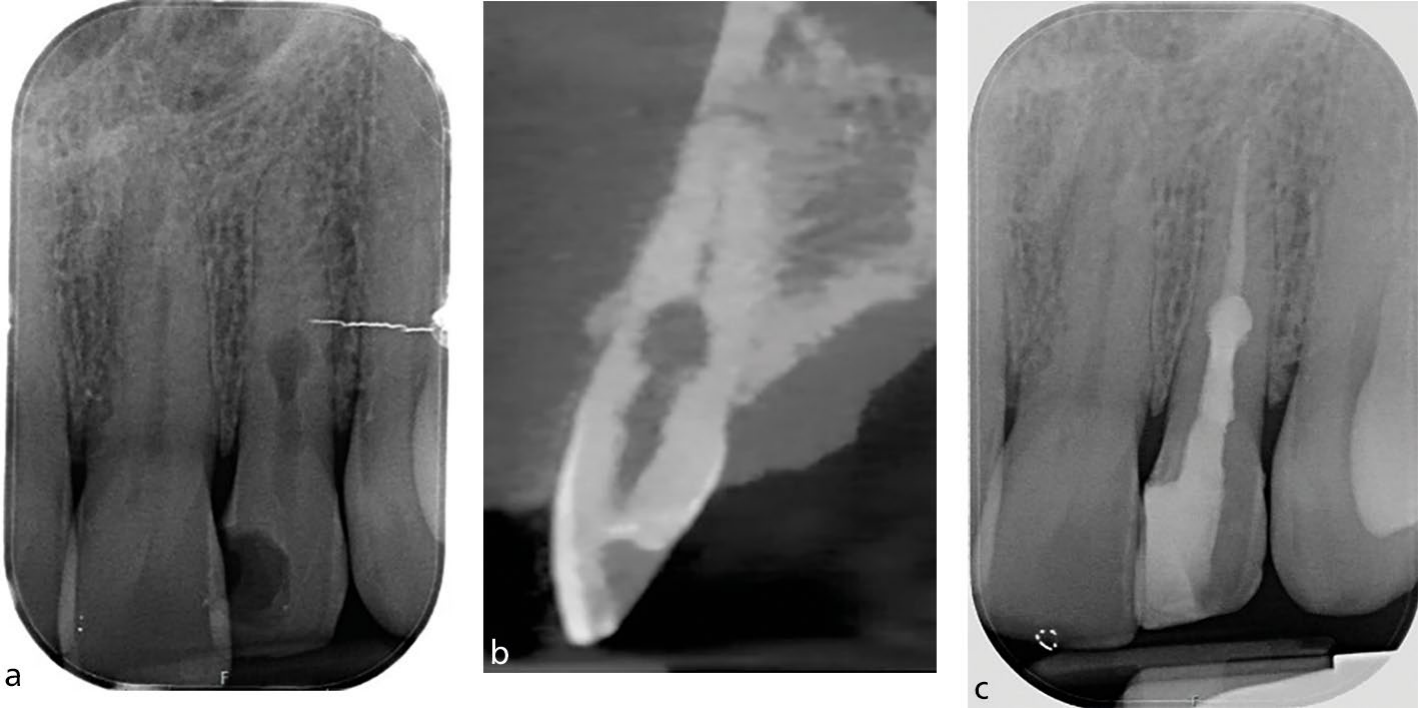
a) IOPA view of the 22 with internal resorption and artefact evident on film; ‘crease'. b) Sagittal view of CBCT section showing the bucco-palatal extent of the lesion and also evidence of canal apical to resorptive lesion. c) IOPA following obturation. Image courtesy of Abdulrahman Mataqi

In order to optimise the diagnostic yield from periapical radiographs, clinicians need to adhere to quality standards as outlined in the European Guidelines.^19^ In brief, this encompasses: i) evidence of optimal image geometry; ii) correct anatomical coverage; iii) good density and contrast; iv) adequate number of radiographs; and v) adequate image processing. All IOPA radiographs should be taken with x-ray holders as routine practice. There are currently three types of intra-oral image receptor which can be used for endodontic imaging: i) dental film (the use of which is decreasing in the United Kingdom); ii) solid-state digital sensors (produce an instantaneous image but can be challenging in patients with limited opening); and iii) photostimulable phosphor plates, commonly used due to the comparative similarity to dental film but are prone to damage from nail marks and creases (Fig. 14). There are also a number of image artefacts in relation to CBCT. These artefacts can interfere with the diagnosis and clinicians should, therefore, be aware of their occurence.^20^ CBCT artefacts include: i) inherent artefacts, arising from the limitation of the process involved in the acquiring of CBCT data, which can be beam geometry of the CBCT, reduced trajectory rotation area and image reconstruction; ii) procedure-related artefacts, for example, misalignment of the x-ray source to the image detectors creating a double contour artefact, similar to patient movement; iii) introduced artefacts resulting in beam hardening (Fig. 11b) and; iv) patient movement, appearing as double contours in the final CBCT image.

IOPA radiographs may be used during endodontic treatment to assist in the following situations: assessing the angulation of access; working length determination in conjunction with electronic apex locators; identification of possible complications; assessment of the canals following completion of instrumentation; and confirmation of appropriate extension of master cones prior to obturation.

The working length is determined by measuring the distance from the coronal reference point to the apical constriction using an electronic apex locator (EAL). There are a number of limitations in determining the working length radiographically, including: additional radiation to the patient; challenges in film positioning (and beam angulation), particularly in patients with exaggerated gag reflex, resulting in distortion, elongation or shortening; lack of 3D representation; superimposition of roots; and lengths based on the radiographic apex rather than the apical constriction with interpretation variability.

An EAL uses two or multiple frequencies to measure the impedance to determine the location of the apical constriction.^21^ EALs have been shown to be more accurate at determining the position of the apical constriction than working length radiographs.^22^ Therefore, when consistent readings are obtained using an EAL, a working length radiograph may not be required, reducing the radiation burden to the patient.

**Fig. 15:**
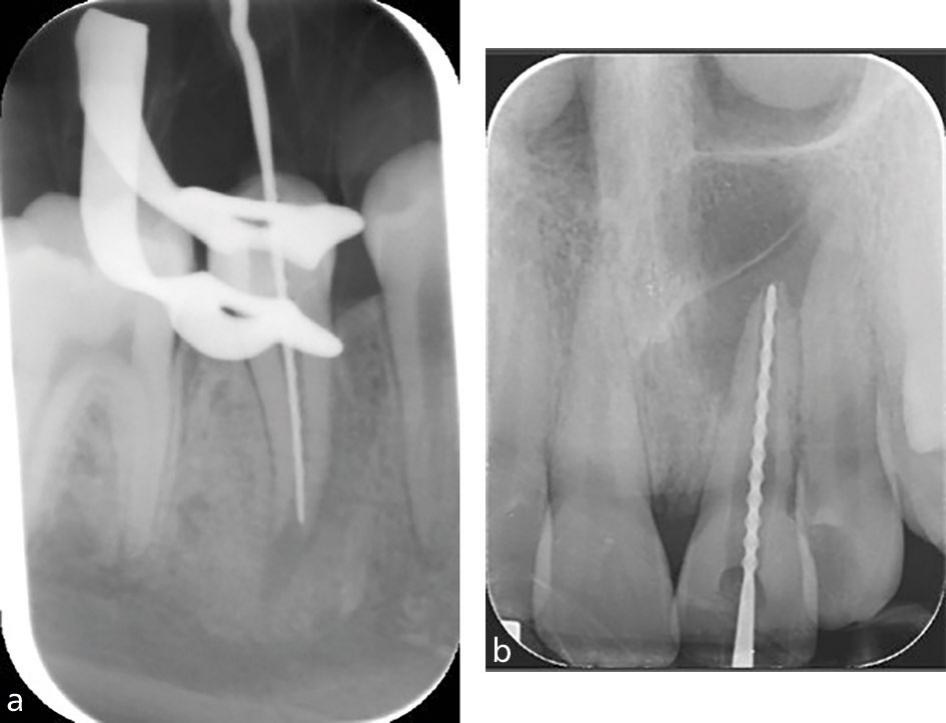
a, b) Working length determination in teeth with loss of apical constriction due to incomplete root development and external inflammatory resorption

**Fig. 16:**
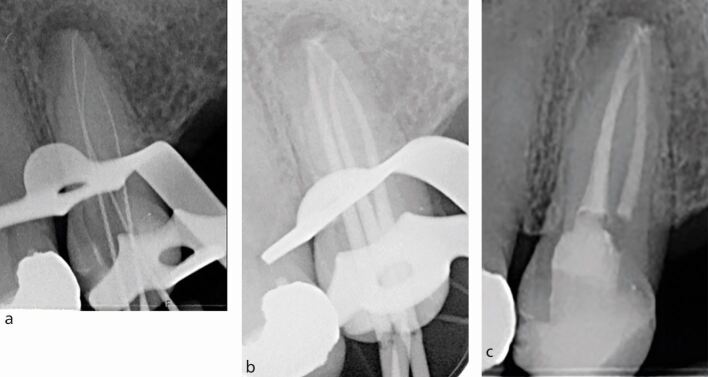
IOPA of upper left first premolar (24) with complex anatomy; three canals. a) Working length radiograph with three size 10k files *in situ* also confirmed with EAL. b) Cone fit radiograph (F1 ProTaper gold matching cones) used to confirm the lengths before final obturation. c) Final obturation (thermoplasticised technique). Image courtesy of Abdullah Alenezi

A radiograph for working length confirmation is recommended when it is not possible to achieve consistent EAL readings, for example, in cases with large or loss of apical constriction due to incomplete root development (Fig. 15a) or resorption (Fig. 15b), in teeth with complex anatomy, and when the apex is in close proximity to important anatomical structures, such as the mental foramen. Figure 16 shows a series of radiographs taken during endodontic treatment of an upper tooth with complex anatomy.

**Fig. 17:**
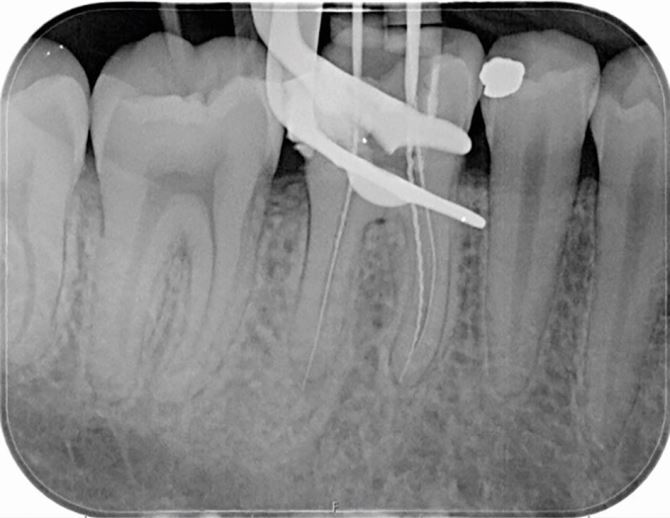
Lower right first molar (46), mesial tube shift allowing visual separation of files in mesial root. Note two different designs of file have been used in the mesial canals to further differentiate between the buccal and lingual canals

In multirooted teeth, a mesial or distal horizontal shift of the x-ray tube can be used when taking a working length radiograph, allowing visual separation of the canals. Furthermore, different cross-sectional design of files can be used to distinguish the differing root canals that may lie in the line of the radiograph (Fig. 17). A tube shift technique can help reduce the number of radiographs taken to determine working length of all root canals, which otherwise must be adequately justified considering the additional radiation burden to the patient.^23^

**Fig. 18:**
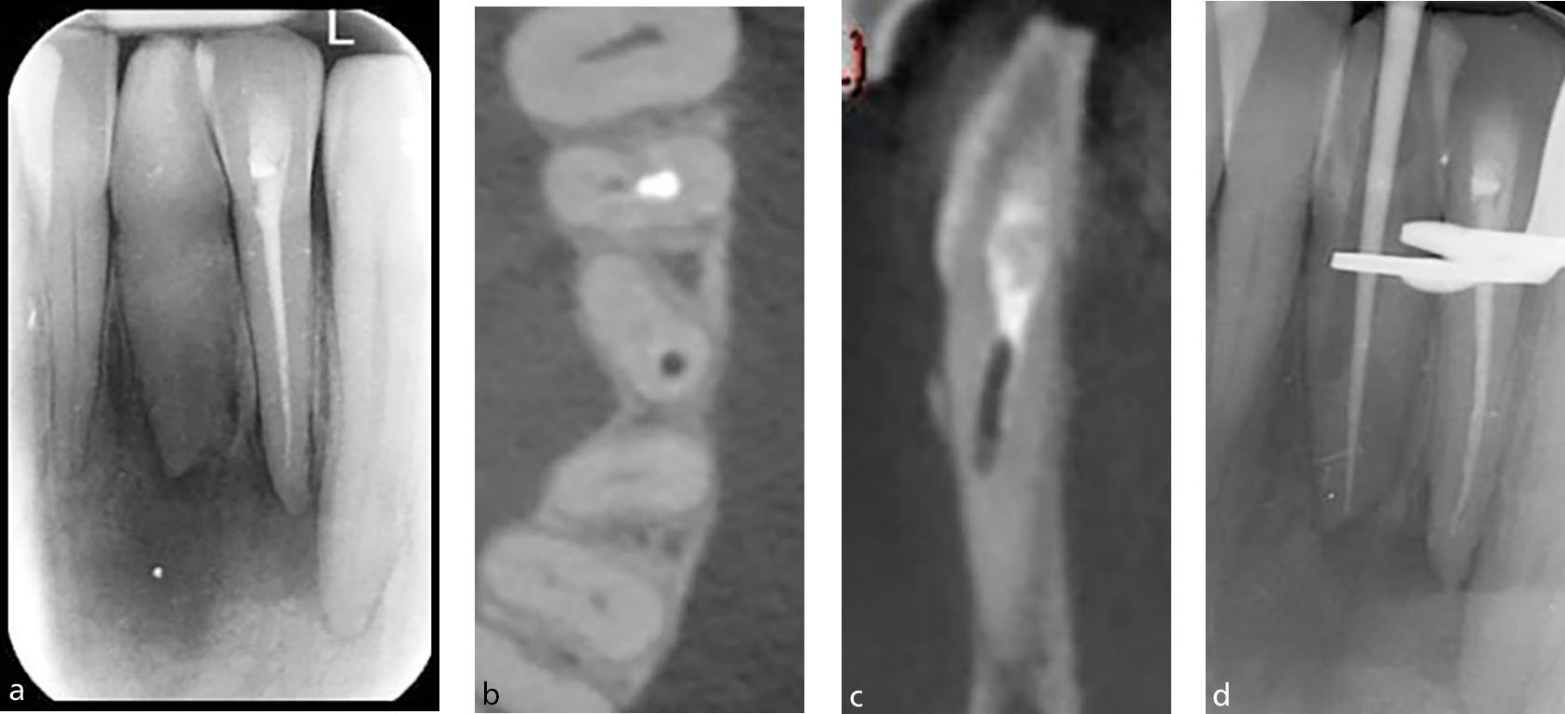
a) IOPA showing a rotated and calcified 31. Following attempted access and troughing with ultrasonics under a dental microscope, a CBCT was taken. b, c) Axial and sagittal views showing a near perforation. This provided adequate information for the clinician to re-align the access to locate the canal. d) Cone fit IOPA showing the canal was successfully re-negotiated

CBCT may also be used during endodontic treatment. For example, CBCT imaging has a higher sensitivity to detect narrow or calcified canals which can assist with location of canals and reorientation of access (Fig. 18). The limit to which a canal can be detected depends on the resolution of the scan, which is proportional to the voxel size. Typically, voxel size can be as low as 0.07 mm, theoretically detecting canals as small as a size 8k file/0.08 mm in diameter.^24^

**Fig. 19:**
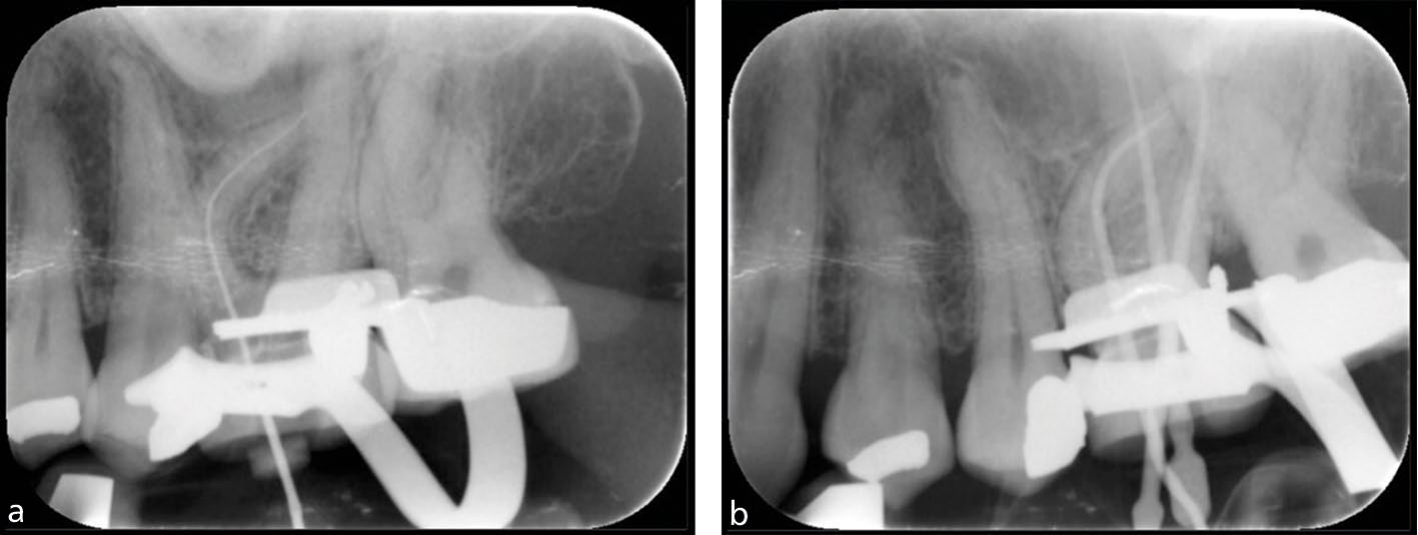
a) Working length radiograph of first mesiobuccal with severe curvature using a 20k file. b) Subsequent master cone radiograph revealing a strip perforation ‘straightening' of the canal following instrumentation with ProTaper Gold F2

A cone fit radiograph is an important additional step in endodontic treatment, which allows the clinician to adjust the length of the cone in a case of under- or over-extension. On occasions, errors in the preparation maybe detected at the cone fit stage (Fig. 19).

**Fig. 20:**
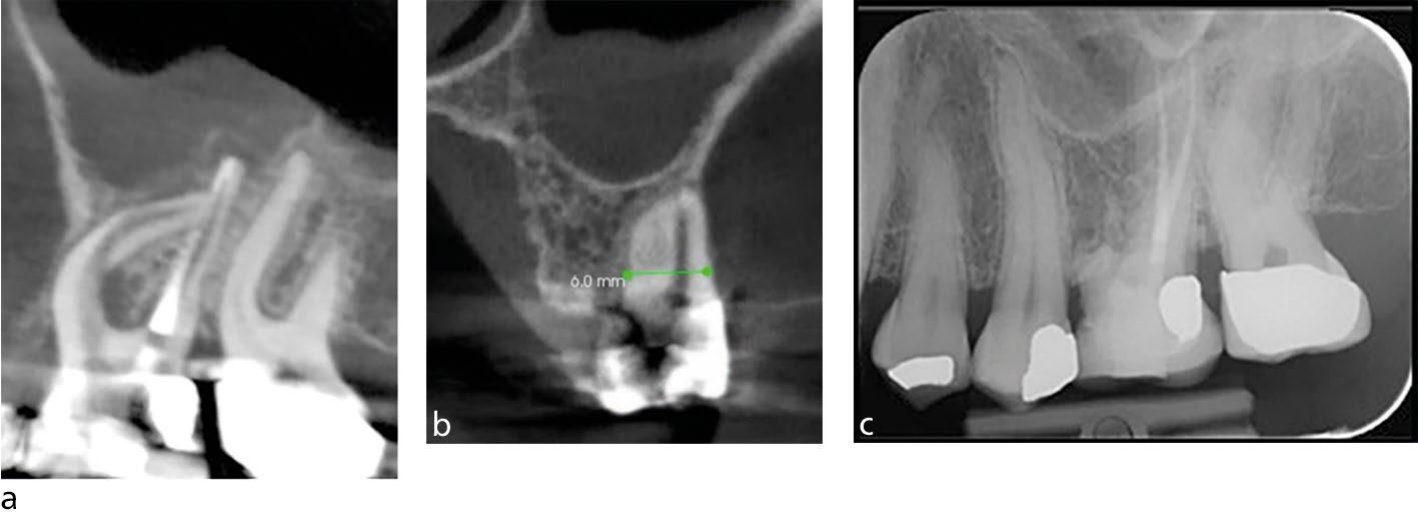
a) Small FOV CBCT showing curved mesio-buccal root with perforation. The scan reveals the proximity of the sinus, adjacent roots, the amount of bone preparation required to expose the mesio-buccal root. b) Depth of resection required for removal. The root was obturated with biodentine and resected surgically. c) Two-year postoperative IOPA radiograph showing signs of bone regeneration

CBCT may be useful in the planning of complex surgical cases, including large lesions and those in close proximity to important anatomical structures.^8^ For example, in multirooted teeth, CBCT can be used to identify and target the roots that are specifically affected with periapical periodontitis (Fig. 20). The extension of the lesion can be clearly identified to assist in planning a flap that will allow full access to the surgical site. Furthermore, proximity of the roots to important anatomical structures, such as the maxillary sinus or mental foramen, can be clearly visualised to assist the clinician in their approach with root end resection safely.

## Dental radiography in the assessment of technical and biologic outcome of root canal treatment

**Fig. 21:**
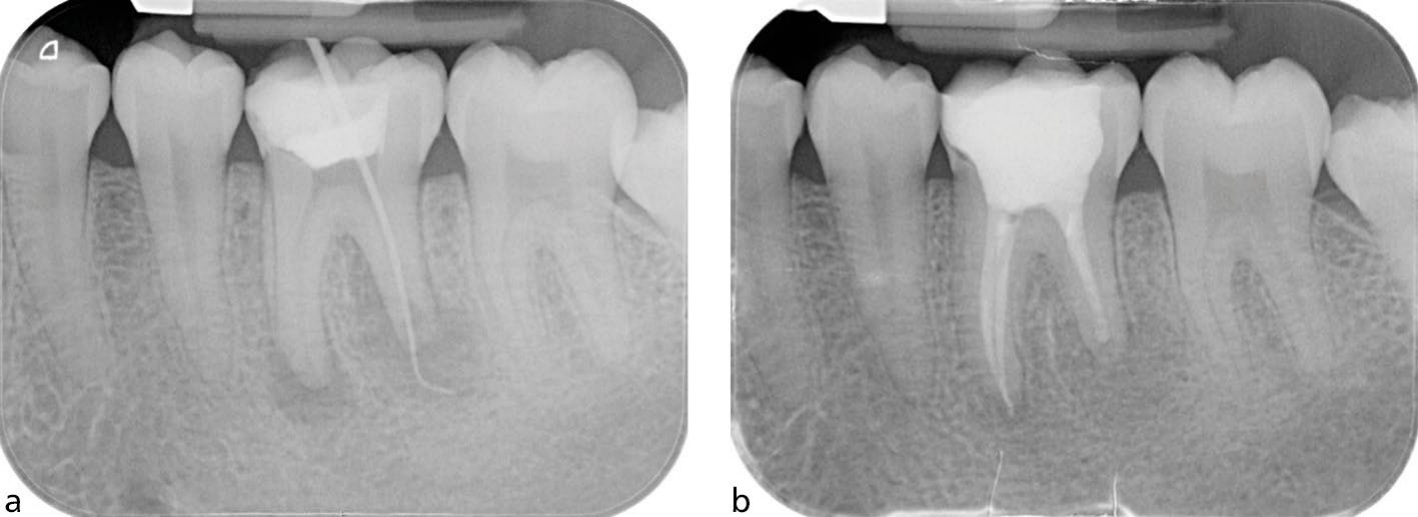
a) IOPA of lower left first molar (36) with a gutta-percha tracer inserted in buccal sinus. Diagnosis: necrotic pulp, symptomatic chronic apical abscess. Considered a high-complexity endodontic case due to inflammatory resorption of the distal root and loss of apical constriction. Periapical radiolucencies present on both mesial and distal root. b) One-year review following endodontic treatment. The distal canal was obturated with mineral trioxide aggregate due to apical gauge ISO 70. IOPA radiograph exposed reveals signs of regeneration of the peri-radicular tissues. Image courtesy of Abdullah Alnaami

**Fig. 22:**
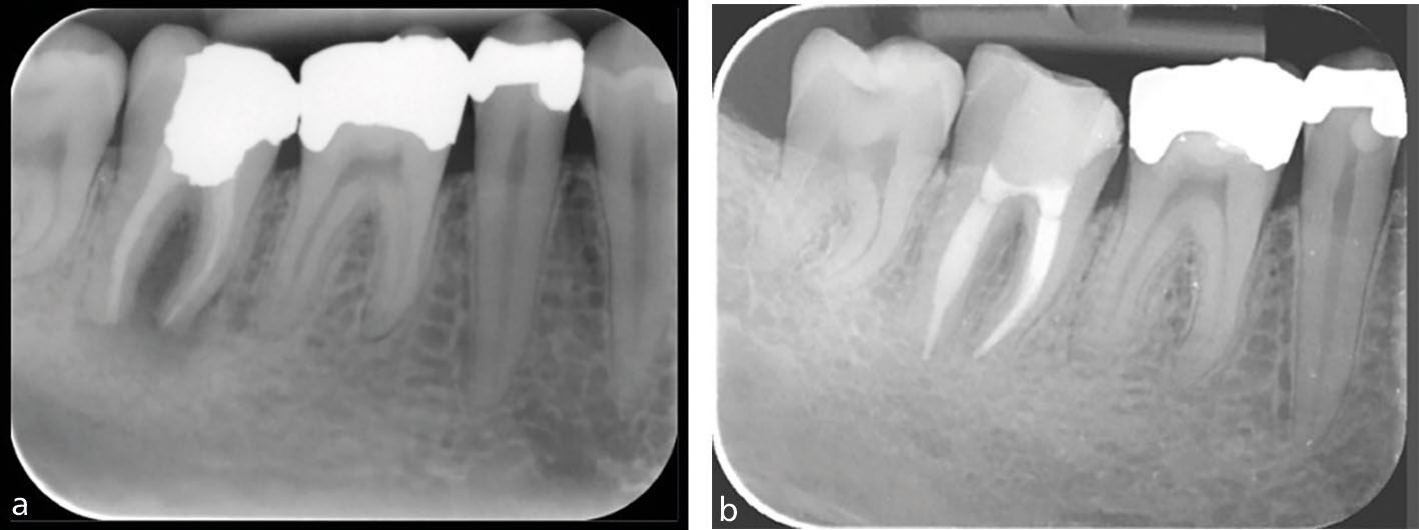
a) IOPA radiograph revealing periapical lesion extending into the furcation area. b) 12-month follow-up IOPA radiograph revealing complete bone regeneration

An IOPA radiograph should be taken immediately following the completion of root canal treatment to assess the technical quality (extension and adaptation) of the root canal filling. Additionally, it provides a baseline image for subsequent monitoring and evaluation of the treatment outcome. The outcome is determined following assessment of patient symptoms and clinical and radiographic examination. The ESE guidelines^25^ recommend that an IOPA is taken annually until complete healing of the periapical tissues, or up to four years where persistent lesions are considered to be associated with post-treatment disease (Fig. 21, Fig. 22).

CBCT is more accurate at detecting the presence of periapical lesions compared to IOPA radiographs.^9^^,^^12^ While CBCT is superior in evaluating the radiographic outcome of endodontic treatment, at present, there is insufficient evidence to advocate its use routinely for monitoring of treatment outcomes. However, its use may be considered in cases where symptoms persist and conventional radiographs fail to yield diagnostic information.^26^ A recent systematic review reported that CBCT was twice as likely to detect periapical lesions than IOPA radiographs and therefore, CBCT should be considered when conventional radiographs are inconclusive.^27^ It has also been shown that CBCT images selected on the basis of the European Commission guidelines developed by SEDENTEXCT project^28^ had a significant impact on diagnosis and treatment planning of the more complex endodontic cases, but CBCT may also have little value in a substantial number of cases.^29^^,^^30^ We have attempted to illustrate the value of CBCT in certain selected endodontic cases, including complex anatomy, difficult diagnoses, pre-surgical planning and ECR cases. A recent systematic review similarly concluded that additional information from CBCT imaging influenced endodontic treatment decision-making in high-difficulty cases.^31^

## The future

Magnetic resonance imaging (MRI) is a non-invasive and non-ionising radiation imaging modality which is currently being researched for use in dentistry.^32^ Dental-dedicated MRI (ddMRI) is a new system which tailors hardware, software and workflow for dentomaxillofacial indications. Earlier signs of inflammation associated with endodontic problems are usually seen within the soft tissues and are therefore detected on ddMRI by demonstrating the increase in fluid contents of the tissue.^33^ The main drawback with ddMRI will be the time taken for the imaging - currently in the region of 15 minutes per patient - and at this stage, ddMRI will only aid in the treatment planning and follow-up of patients who are commencing or have completed endodontic treatment.

Artificial intelligence (AI) is a powerful tool, already used in endodontics, particularly clinical applications such as interpretation of both 2D and 3D images (detecting apical pathoses, cracks and analysis of complex root anatomy). AI algorithms can also be used to help reduce distortion from, for example, metallic restorations^34^ and posts on CBCT scans. At present, however, the use of AI tools requires confirmation of findings from a dental professional.^35^

## Conclusions

The use of imaging is an essential component of endodontic diagnosis and management of cases. The limitations of conventional intra-oral radiographs relative to 3D CBCT imaging have been discussed. Although ‘small field of view' CBCT is now an important technique in the more complex endodontic cases, conventional radiographs remain the standard method of imaging due to accessibility, have a lower radiation dose, and with modern films, have a higher resolution, which are sufficient for diagnosis and the key stages of endodontic treatment in the majority of cases within general dental practice.

## References

[1] Liang Y H, Li G, Wesselink P R, Wu M-K. Endodontic outcome predictors identified with periapical radiographs and cone-beam computed tomography scans. *J Endod* 2011; **37:** 326-331. 10.1016/j.joen.2010.11.032 21329816

[2] Hubar J S, Caballero P. Localization of objects (SLOB Rule). *In* Hubar S, Caballero P (eds) *Fundamentals of oral and maxillofacial radiology*. pp 105-110. New Jersey: Wiley, 2017.

[3] Tai C C, Miller P A, Packota G V, Wood R E. The occlusal radiograph revisited. *Oral Health* 1994; **84:** 47-53. 8779751

[4] UK Government. Ionising radiation: dose comparisons. 2011. Available at https://www.gov.uk/government/publications/ionising-radiation-dose-comparisons/ionising-radiation-dose-comparisons (accessed March 2025).

[5] Ludlow J B, Timothy R, Walker C *et al*. Effective dose of dental CBCT-a meta analysis of published data and additional data for nine CBCT units. *Dentomaxillofac Radiol* 2015; **44:** 20140197. 10.1259/dmfr.20140197 PMC4277438 25224586

[6] Oenning A C, Jacobs R, Salmon B. ALADAIP, beyond ALARA and towards personalized optimization for paediatric cone-beam CT. *Int J Paediatr Dent* 2021; **31:** 676-678. 10.1111/ipd.12797 33844356

[7] Brown J, Jacobs R, Levring Jäghagen E *et al*. Basic training requirements for the use of dental CBCT by dentists: a position paper prepared by the European Academy of DentoMaxilloFacial Radiology. *Dentomaxillofac Radiol* 2014; **43:** 20130291. 10.1259/dmfr.20130291 PMC3887486 24132023

[8] Patel S, Brown J, Semper M, Abella F, Mannocci F. European Society of Endodontology position statement: Use of cone beam computed tomography in Endodontics: European Society of Endodontology (ESE) developed by. *Int Endod J* 2019; **52:** 1 675-1678. 10.1111/iej.13187 31301231

[9] Patel S, Wilson R, Dawood A, Foschi F, Mannocci F. The detection of periapical pathosis using digital periapical radiography and cone beam computed tomography - part 2: a 1-year post-treatment follow-up. *Int Endod J* 2012; **45:** 711-723. 10.1111/j.1365-2591.2012.02076.x 22775142

[10] Bender I B, Seltzer S. Roentgenographic and direct observation of experimental lesions in bone: I, 1961. *J Endod* 2003; **29:** 702-706. 10.1097/00004770-200311000-00005 14651274

[11] Huumonen S, Ørstavik D. Radiological aspects of apical periodontitis. *Endod Topics* 2002; **1:** 3-25.

[12] Patel S, Wilson R, Dawood A, Mannocci F. The detection of periapical pathosis using periapical radiography and cone beam computed tomography - part 1: pre-operative status. *Int Endod J* 2012; **45:** 702-710. 10.1111/j.1365-2591.2011.01989.x 22188219

[13] Nam I, Ryu J, Shin S-H, Kim Y-D, Lee J-Y. Cemento-osseous dysplasia: clinical presentation and symptoms. *J Korean Assoc Oral Maxillofac Surg* 2022; **48:** 79-84. 10.5125/jkaoms.2022.48.2.79 PMC9065647 35491138

[14] Abuabara A, Baratto-Filho F, Aguiar Anele J, Leonardi D P, Sousa-Neto M D. Efficacy of clinical and radiological methods to identify second mesiobuccal canals in maxillary first molars. *Acta Odontol Scand* 2013; **71:** 205-209. 10.3109/00016357.2011.654262 22320229

[15] American Association of Endodontists. AAE Endodontic Case Difficulty Assessment Form and Guidelines. 2022. Available at https://www.aae.org/specialty/wp-content/uploads/sites/2/2022/01/CaseDifficultyAssessmentFormFINAL2022.pdf (accessed March 2025).

[16] Heithersay G S. Clinical, radiologic, and histopathologic features of invasive cervical resorption. *Quintessence Int* 1999; **30:** 27-37. 10323156

[17] Patel S, Foschi F, Mannocci F, Patel K. External cervical resorption: a three-dimensional classification. *Int Endod J* 2018; **51:** 206-214. 10.1111/iej.12824 28746776

[18] Chang E, Lam E, Shah P, Azarpazhooh A. Cone-beam computed tomography for detecting vertical root fractures in endodontically treated teeth: a systematic review. *J Endod* 2016; **42:** 177-185. 10.1016/j.joen.2015.10.005 26631300

[19] European Union. European guidelines on radiation protection in dental radiology: the safe use of radiographs in dental practice. 2004. Available at https://op.europa.eu/en/publication-detail/-/publication/ea20b522-883e-11e5-b8b7-01aa75ed71a1# (accessed March 2025).

[20] Schulze R, Heil U, Gross D *et al*. Artefacts in CBCT: a review. *Dentomaxillofac Radiol* 2011; **40:** 265-273. 10.1259/dmfr/30642039 PMC3520262 21697151

[21] Sayed A, Dighole S, Lobo W M V, Sapkale K, Ramugade M M, Baker D C. Comparative evaluation of the accuracy of six different apex locators in working length determination of molars using intraoral periapical radiographs: an *in vivo* study. *J Conservative Dent Endod* 2024; **27:** 695-700. 10.4103/JCDE.JCDE_189_24 PMC11385914 39262599

[22] Vieyra J P, Acosta J, Mondaca J M. Comparison of working length determination with radiographs and two electronic apex locators. *Int Endod J* 2010; **43:** 16-20. 10.1111/j.1365-2591.2009.01620.x 20002800

[23] Fava L R, Dummer P M. Periapical radiographic techniques during endodontic diagnosis and treatment. *Int Endod J* 1997; **30:** 250-261. 10.1046/j.1365-2591.1997.00078.x 9477811

[24] Spin-Neto R, Gotfredsen E, Wenzel A. Impact of voxel size variation on cbct-based diagnostic outcome in dentistry: a systematic review. *J Digit Imaging* 2013; **26:** 813-820. 10.1007/s10278-012-9562-7 PMC3705012 23254628

[25] European Society of Endodontology. Quality guidelines for endodontic treatment: consensus report of the European Society of Endodontology. *Int Endod J* 2006; **39:** 921-930. 10.1111/j.1365-2591.2006.01180.x 17180780

[26] Karkle A, Slaidina A, Zolovs M *et al*. Comparative analysis of examination methods for periapical lesion diagnostics: assessing cone-beam computer tomography, ultrasound, and periapical radiography. *Diagnostics (Basel)* 2024; **14:** 766. 10.3390/diagnostics14070766 PMC11011571 38611679

[27] Aminoshariae A, Kulild J C, Syed A. Cone-beam computed tomography compared with intraoral radiographic lesions in endodontic outcome studies: a systematic review. *J Endod* 2018; **44:** 1626-1631. 10.1016/j.joen.2018.08.006 30409446

[28] European Union. Cone beam CT for dental and maxillofacial radiology - evidence-based guidelines. 2012. Available at https://op.europa.eu/en/publication-detail/-/publication/ec5936c7-5a29-4a93-9b3a-01a5d78d7b2e (accessed March 2025).

[29] De Almeida F J M, Knutsson K, Flygare L. The effect of cone beam CT (CBCT) on therapeutic decision-making in endodontics. *Dentomaxillofac Radiol* 2014; **43:** 20130137. 10.1259/dmfr.20130137 PMC4082257 24766060

[30] Al-Salehi S K, Horner K. Impact of cone beam computed tomography (CBCT) on diagnostic thinking in endodontics of posterior teeth: a before-after study. *J Dent* 2016; **53:** 57-63. 10.1016/j.jdent.2016.07.012 27461179

[31] Tay K-X, Lim L Z, Goh B K C, Yu V S H. Influence of cone beam computed tomography on endodontic treatment planning: A systematic review. *J Dent* 2022; **127:** 104353. 10.1016/j.jdent.2022.104353 36349644

[32] Niraj L K, Patthi B, Singla A *et al*. MRI in dentistry - a future towards radiation free imaging - systematic review. *J Clin Diagn Res* 2016; **10:** 14-19. 10.7860/JCDR/2016/19435.8658 PMC5121829 27891491

[33] Di Nardo D, Gambarini G, Capuani S, Testarelli L. Nuclear magnetic resonance imaging in endodontics: a review. *J Endod* 2018; **44:** 536-542. 10.1016/j.joen.2018.01.001 29426642

[34] Queiroz P M, Santaella G M, Groppo F C, Freitas D Q. Metal artifact production and reduction in CBCT with different numbers of basis images. *Imaging Sci Dent* 2018; **48:** 41-44. 10.5624/isd.2018.48.1.41 PMC5863018 29581948

[35] Aminoshariae A, Kulild J, Nagendrababu V. Artificial intelligence in endodontics: current applications and future directions. *J Endod* 2021; **47:** 1352-1357. 10.1016/j.joen.2021.06.003 34119562

